# Risk for Emergence of Dengue and Chikungunya Virus in Israel

**DOI:** 10.3201/eid1802.111648

**Published:** 2012-02

**Authors:** Eyal Leshem, Hanna Bin, Uri Shalom, Maayan Perkin, Eli Schwartz

**Affiliations:** The Chaim Sheba Medical Center, Tel Hashomer, Israel (E. Leshem, E. Schwartz);; Tel Aviv University, Tel Aviv, Israel (E. Leshem, E Schwartz); Ministry of Health, Tel Hashomer (H. Bin);; Ministry of Environmental Protection, Jerusalem, Israel (U. Shalom, M. Perkin)

**Keywords:** Emergence, autochthonous transmission, dengue, chikungunya, virus, *Aedes albopictus*, mosquito, mosquito vector, Israel

**To the Editor:** In recent years, *Aedes albopictus*, a mosquito vector of dengue and chikungunya viruses, has rapidly expanded in Europe. Since 2007, the presence of viremic patients with imported cases of dengue and chikungunya virus infection has resulted in several incidences of autochthonous transmission of the viruses in Italy, France, and Croatia (*1**–**4*).

*A. albopictus* mosquitoes have invaded Israel since 2002. A recent national survey showed wide distribution of the mosquito in Israel (*5*), and dengue and chikungunya virus infection are increasingly reported in travelers from Israel who return home from trips to other countries (*6**,**7*). We looked for overlap between the distribution areas of *A. albopictus* mosquitoes in Israel and the living areas of travelers who have returned to Israel with acute dengue or chikungunya virus infections. We discuss the possibility of autochthonous transmission of these viruses in Israel.

All cases of imported, serologically proven acute dengue and chikungunya virus infection registered during 2008–2010 at the National Center for Zoonotic Viruses, Central Virological Laboratory, Israel Ministry of Health, were included in the study. For dengue diagnosis, IgM capture ELISA was run in parallel with dengue indirect IgG ELISA, according to the manufacturer’s (PANBIO, Brisbane, Australia) instructions. For chikungunya diagnosis, microchip technology (Euroimmune, Gross-Groenau, Germany) was performed to detect specific IgM and IgG antibodies. Paired acute- and convalescent-phase samples with a >4-fold increase confirmed an acute case.

Laboratory diagnosis was matched to clinical observations; travel history, along with previous vaccination for yellow fever; and tickborne or Japanese encephalitis. Local infection was differentiated from imported infection by interpretation of a questionnaire that is required from treating physicians before serologic testing. Testing for West Nile virus and Sindbis virus were done to rule out cross-reactions with endemic flaviviruses and alphaviruses, respectively.

Geographic distribution of *A. albopictus* mosquitoes in Israel during 2008–2009 was observed by monitoring the presence of eggs, larvae, and adult mosquitoes in ovitraps and by monitoring reports from municipalities and pest management professionals (*5*). Patients’ areas or municipalities of residence were plotted on a map describing the currently known distribution of *A. albopictus* mosquitoes in Israel. We evaluated the number and proportion of dengue and chikungunya patients in *A. albopictus*–endemic regions.

During the study years, 41 and 15 patients, respectively, received diagnoses of dengue and chikungunya virus infection at the National Center for Zoonotic Viruses (Figure). Of the 41 dengue and 15 chikungunya patients, 27 (66%) and 12 (80%), respectively, lived in areas where *A. albopictus* mosquitoes were endemic (Figure). No autochthonous cases were reported.

**Figure:**
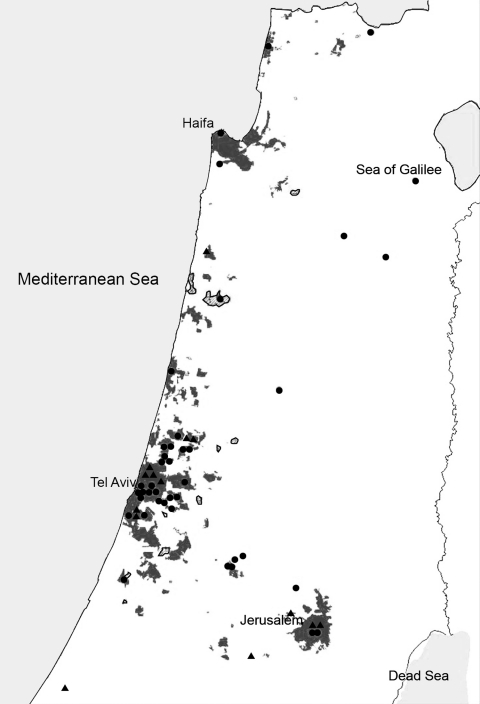
Patients with imported dengue (black circles) or chikungunya (black triangles) virus infection living in *Aedes albopictus*–endemic areas of Israel, 2008–2010. Gray shading indicates known and black outline suspected *A. albopictus*–endemic areas. Of the patients with dengue and chikungunya virus disease, 66% (27/41) and 80% (12/15), respectively, lived in *A. albopictus*–endemic areas.

The establishment of *A. albopictus* mosquitoes in Israel provides suitable conditions for autochthonous transmission of dengue and chikungunya viruses. Although it was traditionally regarded a secondary vector for dengue virus, *A. albopictus* can spread the virus. The potential risk for local outbreaks of dengue or chikungunya virus disease is dependent on the presence of viremic patients and a suitable vector (*8*). Recent reports emphasized the risk for autochthonous transmission of dengue, yellow fever, and chikungunya viruses in Europe. This risk is strengthened by the history of yellow fever and dengue in temperate regions. Dengue virus transmission may follow 2 general patterns: epidemic and hyperendemic dengue. Epidemic dengue transmission may occur as an isolated event when virus is introduced into a region with susceptible hosts and an adequate vector. Such events involve a single virus strain and may manifest in explosive transmission.

Reported cases of autochthonous dengue in France and Croatia were presumed to be related to newly spread *A. albopictus* mosquitoes (*2**–**4*). An outbreak of chikungunya virus infections that occurred in Italy during the summer of 2007 involved >200 persons (*1*). *A. albopictus* mosquito spread in Hawaii was regarded as the cause of a dengue outbreak in 2001 (*9*), and in Florida, dengue fever was recently documented in Key West, where *A. aegypti* mosquitoes are established (*10*).

In this report, we document importation of dengue and chikungunya viruses by travelers to *A. albopictus*–endemic areas in Israel. We show that most patients in Israel with imported dengue and chikungunya virus infection reside in *A. albopictus*–endemic areas of the country. The reported number of serologically proven cases probably underestimates the true extent of the diseases in Israeli travelers because underdiagnosis and underreporting are common. Both dengue and chikungunya virus infection result in viremia that may last up to 5 days, and viremic patients living in *A. albopictus*–endemic areas put the area population at risk for infection.

In summary, we report conditions in Israel suitable for autochthonous transmission of dengue and chikungunya viruses. Although no autochthonous cases have been reported in Israel, they have been reported from other countries where *A. albopictus* mosquitoes are newly endemic. In Israel and other areas where this species is newly endemic, both dengue and chikungunya virus infection should be considered in the differential diagnosis of acute febrile illnesses, even when the patients do not report recent travel to tropical areas. Enhanced surveillance may be needed to prevent epidemic spread of these diseases. Consideration must be taken to isolating suspected (viremic) dengue and chikungunya patients to prevent the establishment of autochthonous transmission.
